# Genetic diversity and population structure analysis of *Forsythia ovata*, a Korean endemic, based on genotyping-by-sequencing

**DOI:** 10.1371/journal.pone.0317278

**Published:** 2025-02-13

**Authors:** Yoo-Bin Lee, Soonku So, Yoo-Jung Park, Halam Kang, Ha-Rim Lee, Jae-Hyeong Kim, Ho-Kwon Gwak, Kyung-Ah Kim, Kyeong-Sik Cheon

**Affiliations:** 1 Department of Biological Science, Sangji University, Wonju, South Korea; 2 Korea National Park Research Institute, Wonju, Korea; 3 Department of Biological Sciences, Kangwon National University, Chuncheon, South Korea; KGUT: Graduate University of Advanced Technology, IRAN, ISLAMIC REPUBLIC OF

## Abstract

The perennial shrub *Forsythia ovata* Nakai, native to the Korean Peninsula, has a highly restricted natural habitat, occurring only in a small area within the Baekdudaegan Mountain Range located in Gangwon-do Province. These characteristics give this species high conservation value, but there is a significant lack of genetic concerning about its populations for conservation purposes. In this study, we utilized genotyping-by-sequencing (GBS) to examine the genetic diversity and population structure of *F. ovata*. Our analysis including 5,017 single nucleotide polymorphisms (SNPs) from 72 individuals, representing nine distinct populations. The results revealed a mean expected heterozygosity (*He*) of 0.212, indicating a moderate level of genetic diversity within the species. Additionally, a relatively low levels of genetic differentiation (*F*_*ST*_) and high gene flow (*N*_*m*_) between populations were detected. The analysis of molecular variance (AMOVA) results indicated that most genetic variation occurred within individuals, accounting for 86.66% of the total variance. In contrast, only 6.90% and 6.44% of the molecular variance was attributed to differences among individuals and between populations, respectively. Considering the results of Bayesian structure analysis on the basis of ∆ *K*, principal coordinate analysis and phylogenetic analysis, we propose two management units for conservation. In addition, given the current conditions faced by *F. ovata*, both in situ and ex situ conservation should be considered for some populations (SG and BD).

## Introduction

The genus *Forsythia* Vahl. belongs to Oleaceae Hoffmanns. & Link. and includes approximately eleven species that are distributed mainly in eastern Asia and eastern Europe [1–5]. Taxa belonging to this genus are characterized as deciduous shrubs that exhibit distyly. This reproductive feature results in two distinct flower types within the species: those with long styles, referred to as ‘pin’ flowers, and those with short styles, known as ‘thrum’ flowers [6,7].

Within this genus of species, *Forsythia ovata* Nakai is endemic to Korea and was first identified on Mt. Geumgangsan and initially described by Nakai[8]. This species is considered closely related to *Forsythia koreana* (Rehder) Nakai and *Forsythia mandschurica* Uyeki owing to its morphological characteristics, but *F. ovata* is distinguished from *F. koreana* in that the stem is erect and the petiole and twig are not hispid. In addition, it is distinguished from *F. mandschurica* in that the hairs are not distributed on the abaxial surface of the leaf, and the heights of the pistil and stamen in pin flowers are not the same [5,6,9,10]. This species is distributed only on the Korean Peninsula [11]. It is listed as an indicator species of limestone areas [12] and a rare plant (not legally protected) [13]. Furthermore, this species is designated Grade V in the list of Floristic Target Species (FT species), which was created to determine conservation priorities for plants on the Korean Peninsula on the basis of their distribution characteristics. This grade is assigned to species that have extremely limited and isolated distributions, are found only in a few areas, and can be considered equivalent to protected species in terms of conservation value [14]. Furthermore, the plant is highly valued in horticulture because of its gorgeous and fragrant flowers. These ecological, distributional, and aesthetic attributes underscore the importance of species in both natural and cultivated settings.

Among endemic plants, species with a very narrow range, such as *F. ovata*, are at greater risk of extinction due to natural or artificial factors, so it is necessary to establish appropriate conservation measures at the national level [15,16]. For more effective conservation strategies to be established, it is necessary to collect taxonomic, ecological, and genetic information on target species [16]. Among these, population genetic information is particularly important because it provides data on gene flow, adaptation ability, and evolutionary history, which can be utilized as crucial information for better understanding the target species and establishing appropriate conservation measures in the future [16–18].

To date, only two studies have examined the genetic diversity of *F. ovata*. One study employed inter simple sequence repeat (ISSR) methods [7], whereas the other utilized allozymes [19]. Notably, these studies yielded conflicting results. An ISSR-based study [7] suggested that *F. ovata* has relatively low genetic diversity, whereas an allozyme-based study [19] indicated high genetic diversity. Given these contradictory findings, it is necessary to conduct further research using more advanced methods to derive accurate results.

Next-generation sequencing (NGS) technology has brought significant changes to various fields of modern biology, including population genetics, and this ability to process vast amounts of genetic data rapidly and at a reduced cost has opened new avenues for scientific inquiry and analysis [20]. The emergence of NGS has led to the development of genotyping-by-sequencing (GBS) technology, which can detect genome-wide single nucleotide polymorphisms (SNPs) with significantly lower error rates and costs than existing technologies can achieve. Owing to these advantages, GBS technology has been widely utilized in recent studies to investigate the genetic diversity of various plant species [21–31].

**Fig 1:**
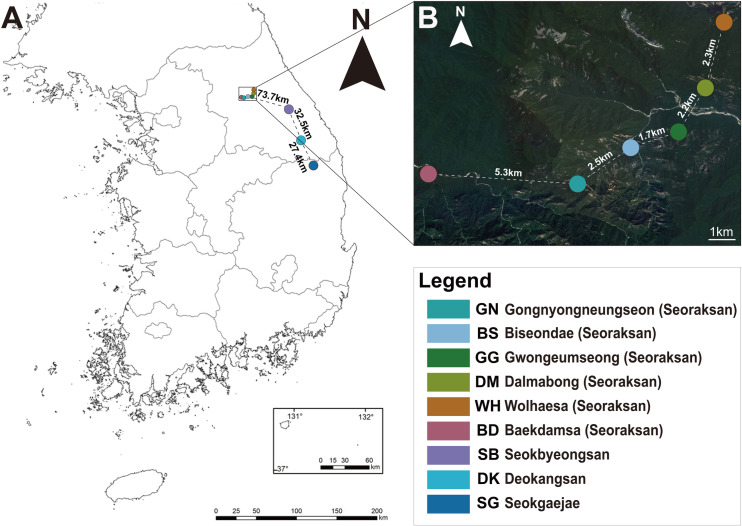
Map of the sampling sites used in this study. (A) Sampling sites for all this study. (B) Enlarged map of sampling sites within Seoraksan National Park (available at https://map.ngii.go.kr/ms/map/nlipCASImgMap.do).

In the present study, we employed the GBS technique to analyze 72 *F. ovata* individuals sampled from nine distinct populations, representing all known natural habitats (Fig 1). Our primary objectives were twofold: (i) to understand the current genetic status by evaluating the genetic diversity and population genetic structure of *F. ovata* and (ii) to provide essential information for establishing appropriate conservation and management strategies for this species.

## Materials and methods

### Plant materials

Since *F. ovata* is not an endangered or protected species, permission was not needed for the collection of plant materials. Distribution information for sampling was confirmed through the literature and specimens stored in herbaria of South Korea. A total of 72 individual plants were collected from nine populations, with 2 to 12 individuals sampled from each population, and samples were collected at intervals of at least 5 m apart to avoid sampling clones (Fig 1 and S1 Table). The voucher specimens for each population were deposited in the Sangji University Herbarium (SJUH).

### DNA extraction, library preparation, and sequencing

Genomic DNA was extracted via the DNeasy Plant Mini Kit (Qiagen Inc., Valencia, CA, USA) following the manufacturer’s instructions. The quality and quantity of the extracted DNA were evaluated with a LabChip GX II (PerkinElmer Inc., Waltham, MA, USA). Samples that passed quality and quantity filters with genomic quality score (GQS) of 3 or higher were subsequently sent to Enzynomics Co. (Daejeon, Korea) for GBS library construction [23]. GBS was following the protocol based on Elshire et al. (2011). DNA was digested with *Ape*KI (Enzynomics co., Daejeon, South Korea), and the ends of the digested genomic DNA were ligated using T4 DNA ligase (Enzynomics co., Daejeon, South Korea) to barcode and common adapters. Then, we pooled adapter-ligated fragments of samples with different barcodes from each individual, and the products were cleaned using HiAccuBead (AccuGene, Incheon, Korea). We enriched the pooled products through PCR to incorporate the primer sequences into the library. After a final clean-up to eliminate small fragments, libraries were visualized on a 1.5% agarose gel and on a fragment Bioanalyzer (Agilent Technologies, California, USA) to determine fragment size distribution. The final GBS libraries were sequenced on one Illumina HiSeq X (Illumina Inc., San Diego, CA, USA) lane per library, which produced 151 bp paired-end reads.

### Sequence analysis, bioinformatics, and SNP identification

The NGSEP Wizard application simplifies genetic analysis by combining multiple processing steps into a single operation. It enables users to generate a complete population variant file directly from raw data, streamlining the typically complex workflow into a user-friendly interface [32]. NGSEP v4.2.1 was used to generate VCF files containing SNP data. Afterward, VCFtools v.4.2 [33] was applied to refine the SNP dataset via the following filtering criteria: SNP missing data ≥  50%, minor allele count ≥  3, minimum quality score <  30, minimum mean depth ≤  3, individuals missing data ≤  50%, SNP data <  95%, minor allele frequency <  5%, minimum mean depth ≤  20, and *p* value for Hardy-Weinberg equilibrium ≤  0.01. Linkage disequilibrium (LD) (*r*^*2*^ >  0.8) was subsequently via Plink v. 1.07 software [34]. The online program PGDSpider v.2.1.1.5 [35] was used to convert the data to different file formats for all downstream analyses described below.

### Data analysis

GenAlEx 6.5 software [36,37] was used to compute the number of alleles (*Na*), the effective number of alleles (*Ne*), Shannon’s information index (*I*), observed heterozygosity (*Ho*), and expected heterozygosity (*He*), as well as to perform AMOVA, estimate gene flow (*Nm*), and conduct PCoA. GenoDive v.2.0b27 [38] was employed to calculate the inbreeding coefficient (*G*_*IS*_). Population genetic structure was analyzed via the Bayesian clustering analysis method conducted in STRUCTURE v2.3.4 software [39]. An admixture model was used to estimate the number of population clusters (*K*) ranging from one to six, and each STRUCTURE run was performed with burn-in sizes of 50,000 and 100,000 Markov chain Monte Carlo (MCMC) iterations with 10 runs per *K* value. The optimal number of genetic groups was determined in two ways: (1) calculating the mean LnP(K) [40] and ∆ *K* [39] as implemented in STRUCTURE Harvester [41] and (2) using StructureSelector [42] to identify the most likely number of clusters present on the basis of the MedMea*K*, MaxMea*K*, MedMed*K* and MaxMed*K* criteria [43]. The population clustering outcomes were processed via the CLUMPAK webserver [44] for alignment and visualization. Genetic relatedness of individuals was assessed via the R packages poppr [45] and adegenet [46] within R version 4.0.4 [47]. A cladogram was subsequently constructed via the UPGMA algorithm in R 4.0.4 [47].

## Results

### SNP analysis and data processing

The sequencing process yielded approximately 700 million reads. After initial quality assessment, the data were refined into a matrix containing 315,584 single nucleotide polymorphisms (SNPs). Initial filtering, which included criteria such as missing SNP data, minor allele count, minimum quality score, and minimum read depth, identified 158,835 SNPs. After the final filtering step, a total of 5017 SNP loci were derived.

### Genetic diversity

The means for the number of different alleles (*Na*) and effective number of alleles (*Ne*) were 1.694 and 1.346, respectively, and the DM population presented the highest *Na* and *Ne* values (1.864 and 1.380, respectively), whereas the SG population, which had the smallest size, presented the lowest values (1.335 and 1.251, respectively). Shannon’s information index (*I*) was highest in the DM (0.378), followed by GN (0.360), BS (0.359), GG (0.354), and so on, with a mean of 0.327. The range of observed heterozygosity (*Ho*) ranged from 0.213 (DK) to 0.253 (GG), whereas the expected heterozygosity (*He*) varied from 0.141 (SG) to 0.240 (DM). Additionally, the inbreeding coefficient (*G*_IS_) was positive in four populations (GN, DM, WH, and DK), whereas five populations (BS, GG, BD, SB, and SG) presented negative values (Table 1).

**Table 1:** Estimates of genetic diversity within *Forsythia ovata* populations.

Pop.	*N*	*Na* ( ± SE)	*Ne* ( ± SE)	*I* ( ± SE)	*Ho* ( ± SE)	*He* ( ± SE)	*G*_IS_
**GN**	11	1.814 (±0.005)	1.366 (±0.005)	0.360 (±0.003)	0.217 (±0.003)	0.230 (±0.002)	0.104
**BS**	7	1.760 (±0.006)	1.373 (±0.005)	0.359 (±0.003)	0.251 (±0.003)	0.232 (±0.002)	-0.007
**GG**	7	1.751 (±0.006)	1.370 (±0.005)	0.354 (±0.003)	0.253 (±0.003)	0.229 (±0.002)	-0.030
**DM**	12	1.864 (±0.005)	1.380 (±0.004)	0.378 (±0.003)	0.249 (±0.003)	0.240 (±0.002)	0.008
**WH**	12	1.775 (±0.006)	1.350 (±0.005)	0.341 (±0.003)	0.220 (±0.003)	0.218 (±0.002)	0.037
**BD**	5	1.589 (±0.007)	1.337 (±0.005)	0.304 (±0.004)	0.237 (±0.004)	0.201 (±0.003)	-0.066
**SB**	7	1.661 (±0.007)	1.345 (±0.005)	0.322 (±0.004)	0.232 (±0.003)	0.210 (±0.003)	-0.028
**DK**	9	1.696 (±0.006)	1.339 (±0.005)	0.323 (±0.004)	0.213 (±0.003)	0.209 (±0.003)	0.041
**SG**	2	1.335 (±0.007)	1.251 (±0.005)	0.205 (±0.004)	0.224 (±0.005)	0.141 (±0.003)	-0.304
**mean**		1.694 (±0.002)	1.346 (±0.002)	0.327 (±0.001)	0.233 (±0.001)	0.212 (±0.001)	

*N*, number of samples; *Na*, number of alleles; *Ne*, effective number of alleles; *I*, Shannon’s information index; *Ho*, observed heterozygosity; *He*, expected heterozygosity; *G*_IS_, inbreeding coefficient; *SE*, corresponding standard error.

### Genetic differentiation and population structure

We performed analysis of molecular variance (AMOVA) to evaluate the degree of genetic differentiation of the nine *F. ovata* populations (Table 2). The analysis results revealed that the majority of genetic variation was within individuals (86.66%), whereas only 6.44% and 6.90% were attributed to differences among populations and among individuals, respectively. The *F*-statistics obtained from the analysis revealed that *F. ovata* was present at a moderate level (*F*_*ST*_ =  0.064, p <  0.001). The analysis revealed a substantial anticipated decrease in overall genetic diversity (*F*_*IT*_ =  0.133, p <  0.001), and the total inbreeding coefficient (*F*_*IS*_ =  0.074, p <  0.001) was calculated as a positive. The pairwise genetic differentiation (*F*_*ST*_) values varied between 0.013 and 0.198, with the lowest value observed between BS and DM and the highest value observed between BD and SG. The estimated gene flow (Nm) ranged from 1.546 (GG and SG) to 7.615 (GN and DM).

**Table 2:** Results of analysis of molecular variance (AMOVA) and significance after 20,000 permutations.

Source	Df	SS	MS	Est. Var.	%	*F*-Statistics	*P* value	Nm
Among Populations	8	10750.091	1343.761	42.936	6.44%	*F*_*ST*_ = 0.064	p < 0.001	3.364
Among Individuals	63	42222.416	670.197	46.053	6.90%	*F*_*IS*_ = 0.074	p < 0.001	
Within Individuals	72	41622.500	578.090	578.090	86.66%	*F*_*IT*_ = 0.133	p < 0.001	
**Total**	**143**	**94595.007**		**667.080**	**100%**			

Df, degrees of freedom; SS, sum of squares; MS, mean of squares; Est. Var., estimated variance; %, percentage of variation; Nm, inferred gene flow.

**Fig 2:**
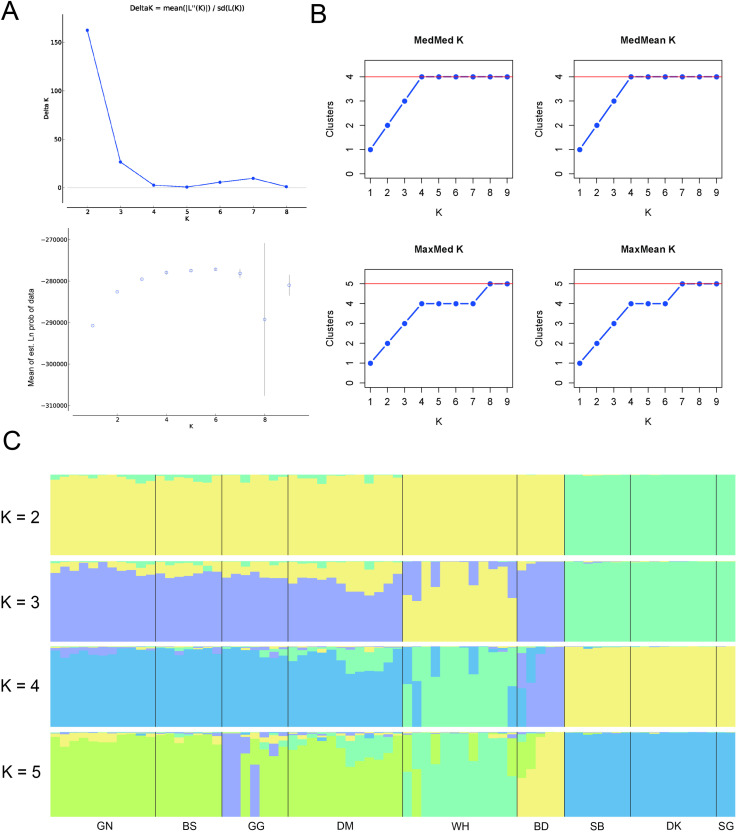
Population structure of 72 *Forsythia ovata* individuals from nine different populations. (A) Relationships between ∆ *K* and *K* obtained through STRUCTURE HARVESTER and the average log-likelihood of *K* against the number of *K* values. (B) The optimal *K* number indicated by the alternative measures MedMed*K*, MedMean*K*, MaxMed*K*, and MedMean*K* applied in StructureSelector and (C) a STRUCTURE bar plot (*K* from 2 - 5). Each bar represents a single individual, and its proportions of assignment to the sample sites are separated by solid black lines.

The peak in the ∆ *K* values indicated that the most likely number of optimal groups was K = 2. Additionally, the likelihood [LnP(D)] increased the most when *K* =  2, and LnP(D) continued to gradually increase as *K* increased (Fig 2A). We evaluated the clustering via four new estimator criteria: ‘MedMed*K*’ (median of medians), ‘MedMean*K*’ (median of means), ‘MaxMed*K*’ (maximum of medians) and ‘MaxMean*K*’ (maximum of means). MedMed*K* and MedMean*K* peaked at *K* =  4, whereas MaxMed*K* and MaxMean*K* had *K* =  5, which was the most likely value (Fig 2B). At *K* =  2, the best number based on ∆ *K*, clear genetic differentiation was observed between the three southern populations (SB, DK and SG) and all other populations. When *K* =  3, the WH showed signs of genetic distinctiveness, separating from the main cluster. At *K* =  4, which corresponded to the peak values of MedMed*K* and MedMean*K*, the BD also emerged as genetically distinct, whereas the other populations maintained their genetic cohesion. At *K* =  5, associated with the maximum values of MaxMed*K* and MaxMean*K*, some individuals (violet) of GG begin to separate from their original group.

**Fig 3:**
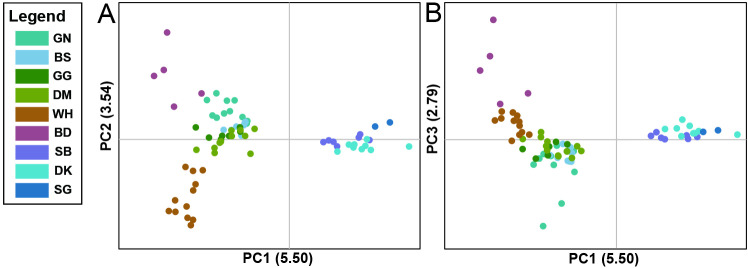
Results of principal coordinate analysis (PCoA) showing axes 1 vs. 2 (A) and 1 vs. 3 (B) for genetic distances between all 72 *Forsythia ovata* accessions.

Principal coordinate analysis (PCoA) results (Fig 3) demonstrated that individuals within the same populations clustered together, revealing patterns of genetic structure consistent with those determined by STRUCTURE analysis. For coordinate 1 (5.50%), the genotypes from six populations located at Seoraksan National Park (GN, BS, GG, DM, WH, and BD) and the remaining three populations were clearly distinguished. For coordinates 2 (3.54%) and 3 (2.79%), genotypes from WH and BD, respectively, showed clear genetic differentiation from the other populations. In addition, genotypes from the four populations located at Seoraksan National Park (GN, BS, GG, and DM) were mixed for all coordinates.

**Fig 4:**
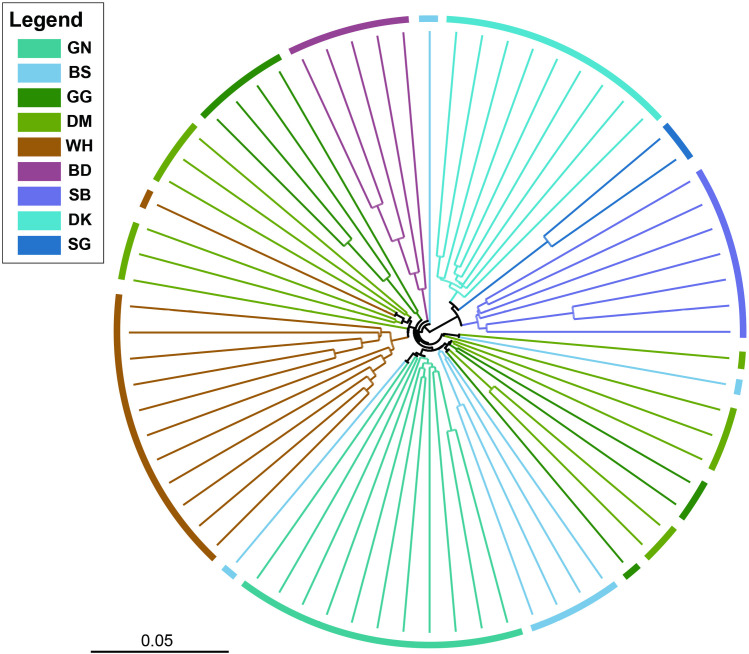
A cladogram of the 72 *Forsythia ovata* accessions from nine populations. Different colors represent different populations.

Phylogenetic reconstruction via the unweighted pair group method with arithmetic mean (UPGMA) algorithm on the 72 genotypes revealed a dendrogram with two distinct clades. The topology of this tree exhibited strong congruence with the population structure patterns identified in the STRUCTURE analysis: the first clade comprised three southern populations (DH, SB, and SC), and the second clade contained six populations located in Seoraksan National Park (GN, BS, GG, DM, WH, and BD). Additionally, the BD genotypes formed a distinct clade, clearly separating from other populations in Seoraksan National Park, whereas genotypes from the remaining five populations formed mixed clades with some or many individuals (Fig 4).

## Discussion

### Genetic diversity in *Forsythia ovata*

The level of genetic diversity within a population is a result of its long-term evolutionary adaptation and demographic history [31,48,49]. Species with greater genetic diversity typically show greater resilience and adaptability to environmental changes, making genetic diversity a key indicator of a species’s potential to survive in fluctuating conditions [31,50,51]. Consequently, accurately assessing and understanding the scope of genetic diversity is essential for crafting effective conservation strategies to protect species.

To assess the genetic diversity of *F. ovata*, we conducted a comparative analysis with previous studies employing similar methodologies. Our findings revealed that, compared with other endemic and woody perennial species, *F. ovata* has relatively low genetic diversity (*He* =  0.212). For example, *Zabelia tyaihyonii* (*He* =  0.233) [31], *Tateraena mongolica* (*He* =  0.348) [52], and *Rhododendron rex* (*He* =  0.54) [48] all demonstrate relatively high levels of genetic diversity. In particular, the genetic diversity of *F. ovata* is lower than that of *Abeliophyllum distichum* [30], which has the same characteristics as *F. ovata*, such as being a Korean endemic species with a limited distribution range and being distylous. In general, species that are distylous or reproduce not only sexually but also asexually are known to have higher genetic diversity than those that do not [7,53–55]. However, in the case of this species, despite having these characteristics, its genetic diversity is considered low. Genetic diversity is related to various factors, such as the life cycle, population size, distribution range, gene flow and mating system [51]. In addition, a reduction in genetic diversity due to genetic drift and inbreeding is known to be closely related to changes in distribution range and population size [50,51,56–58]. *F. ovata* easily declines when the surrounding vegetation grows thick, so currently only a small number of individuals are fragmented and distributed in limited areas such as sunny rocky areas or steep cliffs. Therefore, these ecological factors could be potential causes of low genetic diversity. The genetic diversity measured in a previous study [7] via different methods (ISSRs) was also low (0.243), supporting the results of this study.

Among the populations of *F. ovata*, SG (*He* =  0.141) had the lowest *He* value, followed by BD (*He* =  0.201). The low genetic diversity of the BD is thought to be due to its small habitat area (approximately 30 m^2^) and a small number of individuals (fewer than 20 individuals) (S1 Table). Additionally, BD is the only population located to the west of the Baekdudaegan Mountain Range and is relatively close to the nearest population, GR, only approximately 5 km away in a straight line, but it is isolated by an elevation difference of more than 500 m. In addition, this population is located in a rocky area formed within a valley, so it is continuously disturbed by periodic flooding. These factors are also thought to be responsible for the low genetic diversity in this population.

Meanwhile, in the case of the SG, only 2 individuals were distributed within a natural habitat area of approximately 10 m^2^, and consequently, only these 2 individuals were used in this study. Therefore, due to the potential lack of statistical reliability from the small sample size, we cannot discuss the causes of low genetic diversity in this population. This population was previously relatively large, as indicated by previous studies [7,19] collecting 12 individuals each and estimating the population area to be 500 m^2^, but it is currently the smallest population of *F. ovata*. Therefore, this population has experienced a dramatic decline in size, which is thought to be due to increased canopy coverage in the upper layers and direct destruction of its natural habitat.

### Genetic differentiation and population structure

*N*_*m*_ and *F*_*ST*,_ which are important parameters used to evaluate the genetic structure of populations, are generally known to be negatively correlated [57,59], and this trend was also observed in this study (S1 Fig). The *N*_*m*_ is categorized into three levels: high (≥1.0), medium (0.250–0.99) and low (0.0–0.249). When *N*_*m*_ exceeds 1, it indicates the presence of gene flow between populations [31,52,60]. Additionally, the *F*_*ST*_ values indicate the degree of genetic differentiation among populations, with values between 0 and 0.05 suggesting little to no differentiation, 0.05 to 0.15 indicating moderate differentiation, and 0.15 to 0.25 implying high levels of differentiation. [61]. Despite the large geographical distance, except populations clustered in Seoraksan National Park, our data indicate high levels of gene flow between most population pairs. Moreover, our findings revealed low genetic differentiation between pairs of BS, GG and DM, except between the DM and GG pairs, and high genetic differentiation between the WH and SG pairs and between the SG and BD pairs. All other pairs of populations presented a moderate level of diversity. Flowers of *F. ovata* are entomophilous, i.e., pollinated by insects, and the seeds are spread by gravity [62]. For these reasons, the seeds of this species are not believed to be able to spread far on their own. The high gene flow and relatively low genetic differentiation detected in this study are probably explained by the long lifespan of this species and relatively recent fragmentation. The lifespan of this species is estimated to be 35-40 years, as observed in its close *forsythia* relatives [63], and forests on the Korea Peninsula have been recovering since the 1970s after being mostly destroyed by the Korean War in the 1950s, so most forests are less than 50 years old. Therefore, we believe that although the population became fragmented due to the development of forest canopies with forest recovery, the genotypes are still similar between populations because few generational changes have occurred. However, we expect the *F*_ST_ to increase and the *G*_IS_ to decrease over time.

Genetic variation distribution patterns typically differ between selfing and outcrossing plants. Self-pollinating species maintain most of their genetic variation among populations, whereas outcrossing and long-lived species tend to retain the majority of their genetic diversity within populations [64]. Our AMOVA results (*p* <  0.001) revealed moderate genetic differentiation among *F. ovata* populations. Additionally, the majority of molecular variance was found within individuals rather than among populations, which is consistent with patterns typically observed in outcrossing or long-lived plant species.

The total inbreeding coefficient (*F*_*IS*_ =  0.074) was positive according to the AMOVA results (Table 2), suggesting that a slight degree of inbreeding occurred in *F. ovata*. There are generally no signs of inbreeding in distylous species [30]. In fact, previous studies have shown negative (*F*_*IS*_ =  -0.30) [65] or slightly positive (*F*_*IS*_ =  0.10-0.43) values [66–69], with very strong negative values in the case of *Abeliophyllum distichum*, a Korean endemic shrub similar to *F. ovata*. Lee et al. [30] suggested that population size, morph bias and natural selection can sometimes act as major factors in patterns of genetic diversity among distylous species. We believe that the greatest reason for the high inbreeding coefficient in this species is likely its fragile distribution and the resulting small population sizes that are being maintained. The population fragmentation of many species is related to industrialization, but in this species, it is likely a result of vegetation succession rather than the effects of industrialization. This is because the vegetation between populations is maintained intact because this species is distributed within the Baekdudaegan Mountain Range and national parks, where development activities are strongly restricted by law.

Many studies have employed diverse approaches to assess genetic diversity and population structure [70–74], and Wang [59] argued that integrating three specific analytical methods can lead to more robust outcomes: PCoA, STRUCTURE analysis and UPGMA. The STRUCTURE analyses in this study yielded varying outcomes depending on the evaluation method employed. When the ΔK approach was utilized, the analysis suggested an optimal division of the nine populations into two distinct groups. However, alternative methods produced different results: both MedMed*K* and MedMean*K* indicated a split into four populations, whereas MaxMed*K* and MaxMean*K* suggested a division into five populations. (Fig 2). The Δ*K* method, widely used in structure analysis, efficiently identifies the highest level of population structure and offers an objective criterion for determining the optimal number of genetic clusters. However, it has the disadvantage of potentially underestimating complex hierarchical genetic clusters and being unable to determine the best *K* when the actual *K* is 1 [75]. Conversely, the four newer supervised techniques (MedMed*K*, MedMean*K*, MaxMed*K*, and MaxMean*K*) excel at detecting fine-scale and hierarchical genetic structures, especially when prior population information is available. However, they require more computational resources, can be sensitive to data noise, and may sometimes overestimate cluster numbers [76]. In this study, the results of PCoA and UPGMA provided stronger support for the ΔK results. These findings provide highly valuable information for identifying more appropriate management units when formulating conservation strategies for this species.

### Implications for the conservation of *F. ovata*

For rare and endangered plants, understanding genetic variation patterns within and among populations is essential because maintaining genetic diversity is crucial for species adaptive potential in changing environments. Moreover, this fundamental knowledge is essential to developing effective conservation strategies and management plans, ultimately supporting the long-term survival of these vulnerable species. [31,77,78]. Our findings suggest that *F. ovata* has a very low adaptive capacity to cope with changing environmental conditions. We believe that the low genetic diversity of *F. ovata* is due to natural phenomena. Specifically, a bottleneck could have occurred due to direct population decline driven by interspecific competition accompanying the vegetation succession process, lowering the genetic diversity of this species. To preserve this species in its natural habitat, therefore, human intervention is necessary. Such interventions may include thinning the overhead vegetation and removing nearby competing plants. This poses a dilemma between the protection of rare plants and the artificial destruction of vegetation that is well developed or already in the recovery stage. Sixty-five percent (approximately 6,300,000 ha) of the total area of South Korea is forest with well-developed and preserved vegetation [79], and *F. ovata* is distributed in only a very small portion of this area. In addition, *F. ovata* is a species with very high conservation value because this species is a Korean endemic that will become extinct throughout the world if it becomes extinct in Korea. We believe that rather than allowing these small areas of forests to undergo natural forest succession over time, it would be more valuable to apply artificial measures to ensure that plants with high conservation value can continue to grow. If aggressive methods of artificially modifying the vegetation of native habitats are chosen as a conservation strategy for this species, various ecological studies involving the type of vegetation preferred by the species and the degree of upper canopy development should be conducted and then systematically implemented on the basis of scientific evidence.

Efforts to preserve this species should prioritize protecting populations at the trailing edge of its distribution range. This is because their prolonged isolation, successful adaptation, and persistence imply a significant capacity for evolutionary adaptation, possibly playing a crucial role in the long-term conservation of genetic diversity and phylogenetic history of plant species [30,31,80]. In this context, we believe that the SG merits particular consideration because its location is at the southernmost limit of the distribution range. In addition, this population is at a very high risk of extinction, with only two individuals remaining due to rapid population decline due to an increased vegetation canopy and habitat destruction. Fortunately, the diverse findings from this study, including the results from PCoA, STRUCTURE analysis and the UPGMA technique (Figs 2–4), revealed that this population had genotypes similar to those of the adjacent populations DK and SB, so it is believed that good results can be obtained if a conservation and restoration strategy including these populations is established. Additionally, since the area where the SG is located is considered to have very unfavorable conditions for the growth of *F. ovata*, the genotype will need to be maintained through ex situ conservation strategies until environmental improvements are made through vegetation removal. The BD is also considered an important population because of its unique geographic location to the west of the Baekdudaegan Mountain Range. As mentioned above, this population is affected by continuous flooding, which is a very serious threat that could lead to sudden population extinction. Therefore, we suggest that the unique genotypes of this population can also be maintained continuously through ex situ conservation.

## Conclusion

This study investigated the population genetics of *F. ovata* via 5,017 high-density single nucleotide polymorphisms (SNPs) derived from 72 individuals across nine populations. The low genetic diversity of this species is thought to be due to the increase in the upper canopy caused by vegetation succession. Additionally, the relatively low genetic differentiation and high gene flow observed in the present study were likely due to the long lifespan and relatively recent fragmentation of this species. To preserve this species naturally in situ, anthropogenic efforts, including reducing the vegetation canopy and directly removing competing plants, are needed. Additionally, SG and BD, which are distributionally important populations, require separate ex situ conservation efforts to maintain their genotypes. This study is the first to perform population genetic analysis of *F. ovata* via GBS. The insights gained from our analysis, along with our proposed conservation strategies, provide crucial insights for the preservation and management of this species.

## Supporting information

S1 Fig Genetic differentiation coefficient *F*_*ST*_ (below diagonal) and gene flow *N*_*m*_ (above diagonal) between populations (TIF)

S1 Table The sampling information for nine populations of *F. ovata.* (DOCX)

S1 File SNP genotype information in variant calling format (vcf) for 72 samples of *F. ovata.* (XLSX)
