# Supplementary figures and images for: Genetic diversity and population structure analysis of Forsythia ovata, a Korean endemic, based on genotyping-by-sequencing
Source: PLoS One. 2025 Feb 13;20(2):e0317278. doi: 10.1371/journal.pone.0317278 (PMC11825039; doi:10.1371/journal.pone.0317278)

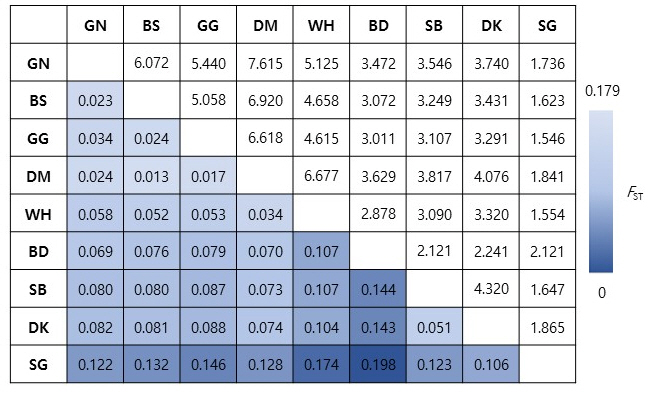

Supplement: S1 Fig — (TIF) [file pone.0317278.s001.tif]
