# Supplementary material for: Genetic diversity and population structure analysis of Forsythia ovata, a Korean endemic, based on genotyping-by-sequencing
Source: PLoS One. 2025 Feb 13;20(2):e0317278. doi: 10.1371/journal.pone.0317278 (PMC11825039; doi:10.1371/journal.pone.0317278)
Supplement: S1 Table — (DOCX) [file pone.0317278.s002.docx]

Table S1. The sampling information of nine population of *F. ovata*.

| **Population** | **Number of Individuals** | **Approximately**  **Populaion size (m^2^)** | **Locality** | **Coordinates** | **Elevation** |
| --- | --- | --- | --- | --- | --- |
| **GN** | 11 | 350 | Gongnyonneungseon,  Sokcho-si Gangwon-do | N38.15540 E128.44773 | 1090m |
| **BS** | 7 | 250 | Biseondae,  Sokcho-si, Gangwon-do | N38.16281 E128.46975 | 338m |
| **GG** | 7 | 200 | Gwongeumseong,  Sokcho-si, Gangwon-do | N38.16296 E128.48572 | 700m |
| **DM** | 12 | 500 | Dalmabong,  Sokcho-si, Gangwon-do | N38.17889 E128.51183 | 444m |
| **WH** | 12 | 400 | Wolhaesa,  Sokcho-si, Gangwon-do | N38.19953 E128.50776 | 160m |
| **BD** | 5 | 30 | Baekdamsa,  Inje-gun, Gangwon-do | N38.15579 E128.38695 | 500m |
| **SB** | 7 | 250 | Mt. Seokbyeong,  Gangneung-si, Gangwon-do | N37.58763 E128.89448 | 997m |
| **DK** | 9 | 300 | Mt. Deokang,  Samcheok-si, Gangwon-do | N37.31894 E129.01383 | 610m |
| **SG** | 2 | 10 | Seokgaejae,  Samcheok-si, Gangwon-do | N37.08557 E129.14089 | 830m |
